# The role of hydroxyindoles in protecting neuronal cultures from ferroptosis

**DOI:** 10.1038/s41420-025-02608-4

**Published:** 2025-07-16

**Authors:** Md. Jakaria, Jason R. Cannon

**Affiliations:** 1 School of Health Sciences, Purdue University, West Lafayette, IN 47907 USA; 2 Purdue Institute for Integrative Neuroscience, Purdue University, West Lafayette, IN 47907 USA

**Keywords:** Cellular neuroscience, Stress signalling

## Abstract

Hydroxyindoles are organic compounds characterized by a hydroxyl group attached to an indole ring. One notable example is 5-hydroxyindole, which can be found in humans, plants, and microorganisms. The structure of 5-hydroxyindole is integral to molecules such as melanin, serotonin and 5-hydroxyindoleacetic acid (a serotonin metabolite). Ferroptosis is a regulated form of cell death driven by uncontrolled phospholipid peroxidation, which has been linked to the pathogenesis of neurodegenerative diseases, including Alzheimer’s and Parkinson’s. The impact of hydroxyindoles on ferroptosis remains largely unexplored. This study tests the hypothesis that different hydroxyindoles can modulate ferroptosis in neuronal cultures through specific structure-activity relationships. We used various pathway-specific inducers, including erastin, RSL3, and FINO2, to induce ferroptosis. Cytotoxicity was evaluated using calcein AM, MTT (thiazolyl blue tetrazolium bromide), and LDH (lactate dehydrogenase) release assays. Glutathione levels were measured with the monochlorobimane assay, and intracellular ATP (adenosine triphosphate) levels were quantified using the ATP-Glo™ Bioluminometric cell viability assay. We also performed the ABTS (2,2’-azino-bis(3-ethylbenzothiazoline-6-sulfonic acid)) assay to evaluate the radical-trapping antioxidant activity of the compounds. Our findings indicate that hydroxyindoles function as a class of ferroptosis inhibitors in cell cultures. Among the hydroxyindole analogs studied, 3-hydroxyindole emerged as the most potent inhibitor of ferroptosis in both HT-22 (mouse hippocampal neurons) and N27 (rat dopaminergic neurons) cell lines. In contrast, 5-hydroxyindole and its specific analogs, such as serotonin and 5-hydroxyindoleacetic acid, were found to be less effective in inhibiting ferroptosis in HT-22 cells. Further investigations into the underlying mechanisms revealed that hydroxyindoles inhibit ferroptosis through their intrinsic radical-trapping antioxidant activity. In conclusion, several hydroxyindole analogs, including 3-hydroxyindole, 6-hydroxyindole, and 7-hydroxyindole, have been identified as inhibitors of ferroptosis, highlighting their potential as therapeutic agents for conditions involving neuronal loss caused by ferroptosis.

## Introduction

**Fig. 1:**
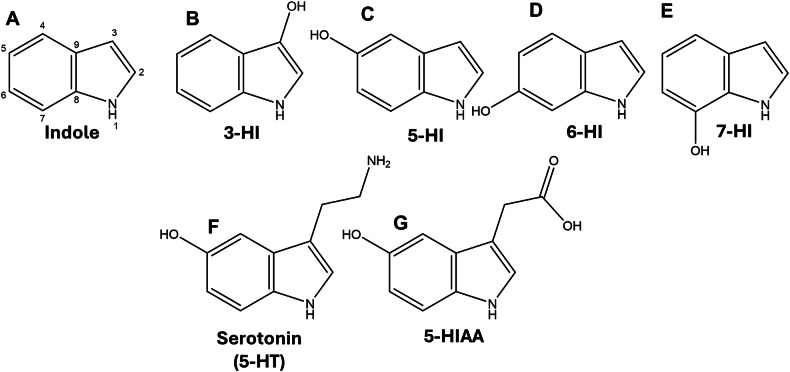
Chemical structures of the hydroxyindole analogs that were tested in this study. The indole structure consists of a benzene ring and a pyrrole ring (a five-membered ring with a nitrogen atom) (**A**). Altering the position of a hydroxyl group on the indole ring results in several hydroxyindole compounds, including 3-, 5-, 6-, and 7-HI. (**B**–**E**). Introducing additional groups to 5-HI results in either serotonin or the serotonin metabolite 5-HIAA (**F**&**G**).

Hydroxyindoles are a class of organic compounds characterized by a hydroxyl group attached to an indole ring (Fig. 1A). These compounds occur naturally and are used to synthesize various medicinal drugs. One notable analog of hydroxyindole is 5-hydroxyindole (5-HI), which can be found in humans, plants, and microorganisms [1, 2].

In the gut, microbiota that express tryptophanase can convert L-tryptophan into 5-HI. The enzyme tryptophanase transforms L-tryptophan into both 5-hydroxy-L-tryptophan and indole as intermediates; subsequently, 5-hydroxy-L-tryptophan is converted into 5-HI [1]. The structure of 5-HI is essential for the production of several biologically active molecules in humans, including melanin—the natural pigment responsible for skin, eye, and hair color—and serotonin (5-hydroxytryptamine; 5-HT), a neurotransmitter and hormone essential for regulating various bodily functions such as mood and sleep. The major metabolites of serotonin, including 5-hydroxyindole acetic acid (5-HIAA) and N-acetylserotonin, also contain the 5-HI structure.

Growing evidence suggests that 5-HI and its analogs show a protective role against various forms of cellular stress [3–9]. For example, 5-HI has been shown to defend against oxidative stress, apoptosis, and mitochondrial dysfunction induced by tert-butylhydroperoxide in human fibroblast cells [3]. Several independent studies have also found that 5-HI analogs, such as serotonin and N-acetylserotonin, protect against inflammation, mitochondrial stress, oxidative stress, apoptosis, and activated autophagy [7–9], thereby safeguarding against neuronal damage. Another analog of 5-HI, N-[2-(5-hydroxy-1H-indol-3-yl)ethyl]-2-oxopiperidine-3-carboxamide (HIOC), also a derivative of N-acetylserotonin, has been found to activate tyrosinase kinase B (TrkB) receptor signaling [10], protecting the retina from light-induced degeneration [10].

Recent studies indicate that serotonin metabolism is involved in ferroptosis, a regulated form of cell death characterized by oxidative stress and uncontrolled phospholipid peroxidation [11–14]. Ferroptosis has been identified as a potential mechanism underlying various degenerative conditions, including neurodegenerative diseases [15, 16]. Serotonin has been shown to inhibit ferroptosis in different cancer cells. This effect is attributed to serotonin’s ability to be taken up by cells and its function as a radical-trapping antioxidant (RTA) [11, 13]. Furthermore, the serotonin receptor 2B (5-HT2B) inhibits ferroptosis in gastric cancer cells and enhances viability under metabolic stress, which can lead to increased tumor growth and reduced patient survival [12]. Monoamine oxidase A (MAO-A) is an enzyme that breaks down monoamine neurotransmitters, including serotonin. Research has shown that MAO-A diminishes the protective effects of serotonin against ferroptosis. This diminution occurs because MAO-A degrades serotonin. As a result, cancer cells deficient in MAO-A may resist ferroptosis when treated with serotonin [11].

In addition to serotonin, N-acetylserotonin has been shown to protect from ferroptosis-associated injuries. Studies indicate that N-acetylserotonin mitigates damage related to ferroptosis caused by traumatic brain injury and hypoxia in rodent models [14, 17]. Beyond the analogs above, another hydroxyindole analog, 3-HI, has demonstrated protective effects against toxicity induced by amyloid beta in PC12 pheochromocytoma cells [4].

While hydroxyindoles protect against ferroptosis, the precise role of these molecules in this process remains unclear. It is uncertain whether various hydroxyindole analogs, such as 3-, 5-, 6-, and 7-HI, and 5-HIAA (Fig. 1B–G), influence ferroptosis. Furthermore, it is undetermined if 5-HI is more effective than other hydroxyindoles in this context. To address this significant gap in the literature, we investigated the hypothesis that individual hydroxyindoles modulate ferroptosis in neuronal cultures through structure-activity relationships.

## Study methodology

### Reagents and chemicals

RPMI 1640 (Cat#MT10041CM) from Corning Inc, USA; Penicillin and streptomycin (Cat#15140122), fetal bovine serum (FBS; Cat#A5256801) and hanks’ balanced salt solution (HBSS; Cat#14-175-079) from Gibco, a part of Thermo Fisher Scientific, Waltham, MA, USA; Calcein AM (Cat#C1430) and BODIPY™ 581/591 C11 (Cat#D3861) from Invitrogen, also a part of Thermo Fisher Scientific; Ammonium iron (II) sulfate hexahydrate (Iron; Cat#201370250), 2,2’-Azino-bis(3-ethylbenzothiazoline-6-sulfonic acid) diammonium salt (ABTS; Cat#J65535.03) and potassium persulfate (Cat#202010250) from Thermo Fisher Scientific; Liproxstatin 1 (Lip-1; Cat#S7699), and RSL3 (Cat# S8155) were from Selleck Chemicals LLC, Houston, TX, USA; ATP-Glo™ Bioluminometric Cell Viability Assay kit (Cat#30020-2) from Biotium, Inc, CA. USA; Cytotoxicity Detection KitPLUS (LDH; Cat#4744926001) from Roche; FINO2 (Cat# CAY25096) was obtained from Cayman Chemical, Michigan, USA; 3-HI (Cat#sc-490580), 5-HI (Cat#sc-254834), 6-HI (Cat#sc-254886), and 7-HI (Cat#sc-217454) were obtained from Santa Cruz Biotechnology, Inc, Dallas, Texas, USA; Erastin (Cat#329600), rotenone (Cat#R8875), thiazolyl blue tetrazolium bromide (MTT; Cat#M2128), IGEPAL® CA-630 (Cat#I8896), glutathione s-transferase (Cat#G6511), monochlorobimane (mBCI; Cat#69899), serotonin (Cat#H9523) and 5-HIAA (Cat#H8876) was obtained from Sigma-Aldrich Pty Ltd, an affiliate of Merck KGaA, Darmstadt, Germany

### Cell culture

The study was conducted on two immortalized cell lines. The HT-22 mouse hippocampal cell line was subcloned from the HT-4 cell line, which was generously provided by Dr. Val J. Watts, Purdue University, USA. The N27 rat dopaminergic neural cell line was derived from E12 rat mesencephalic tissue, supplied by Dr. Wei Zheng and Dr. Jean-Christophe (Chris) Rochet from Purdue University, USA; however, the original stock of the N27 cell line was obtained from Dr. Curt Freed at the University of Colorado, USA. These cell lines were cultured in RPMI 1640 media, supplemented with 10% fetal bovine serum and 1% penicillin-streptomycin. All cell-based assays were conducted in RPMI 1640 media with the same supplements.

### Cell viability and cellular death assays

Multiple independent measures in this study assessed cell loss. The MTT and calcein AM assays were utilized to measure viable cells and evaluate cytotoxicity. The MTT assay is based on the capacity of nicotinamide adenine dinucleotide phosphate (NADPH)-dependent oxidoreductase enzymes and/or succinate dehydrogenase in the mitochondria of metabolically active cells to convert the yellow MTT dye into purple formazan crystals. Consequently, metabolic changes might affect the MTT signal without necessarily causing cell death; however, during acute ferroptosis, a consistent correlation between MTT results and those from other viability assays has been noted, indicating that any metabolic influence is likely minimal [18]. In contrast, the calcein AM assay utilizes calcein AM, a non-fluorescent, hydrophobic compound that readily passes through the membranes of live cells. Once inside the cell, intracellular esterases convert calcein AM into calcein, a hydrophilic compound that fluoresces strongly and remains in the cytoplasm. The level of fluorescence measured in a sample is directly proportional to the number of live cells present.

In the MTT and calcein AM experiments, cells were seeded at a density of 1.5 × 10^4^ cells per well in 96-well plates. The cells were then co-treated with the tested compounds and ferroptosis inducers for 24 h. For the MTT assay, 20 μL of MTT solution (10 mg/mL) was added to each well and incubated for 2–3 h. After incubation, the media was aspirated from each well, and DMSO was added to dissolve the formazan crystals formed in the viable cells. The absorbance of the dissolved formazan was measured at 570 nm using a Molecular Devices SpectraMax M2e Multi-Mode Microplate Reader.

For the calcein AM assay, following treatment, the cells were washed with HBSS buffer and incubated in a diluted solution of calcein AM in HBSS, resulting in a final concentration of 1 µM. This incubation lasted 30 min to 1 h in a cell culture incubator. Cell viability was then measured fluorometrically using the same Molecular Devices SpectraMax M2e Multi-Mode Microplate Reader, with an excitation wavelength of 494 nm and an emission wavelength of 517 nm. The percentage of viable cells, determined by either the MTT or calcein AM assay, was calculated relative to the control cells.

We assessed cytotoxicity-driven cell death using the lactate dehydrogenase (LDH) release assay with the Cytotoxicity Detection Kit, as described in a previous article [19]. This assay measured the amount of LDH released into the culture supernatants due to increased permeability associated with cell death. Cells were seeded and treated according to the cell viability assay protocol for an experiment. Following treatment, 100 µL of the supernatant was transferred into a 96-well plate and mixed with 100 µL of freshly prepared assay reagent, which included a catalyst and dye solution. The mixture was then incubated in the dark at room temperature for 30 min. After incubation, the colorimetric absorbance was read at a sample wavelength of 492 nm and a background wavelength of 600 nm using a Molecular Devices SpectraMax M2e Multi-Mode Microplate Reader. Cells treated with DMSO released LDH spontaneously, while cells treated with Triton X-100 exhibited maximum LDH release. The percentage of LDH release was calculated using the following formula:

% of LDH release = (experimental value – spontaneous release)/(maximum release – spontaneous release) × 100.

### ATP-Glo™ bioluminometric cell viability assay

The ATP-Glo™ Bioluminometric Cell Viability Assay was used to measure the levels of adenosine triphosphate (ATP) in cells. ATP is the primary energy source for cellular functions, including metabolism. This detection kit utilizes the ability of firefly luciferase to use ATP for the oxidation of D-Luciferin, resulting in light production. The amount of light generated correlates with the amount of ATP present, which indicates the presence of metabolically active cells. Thus, the assay allows for determining viable cell numbers based on the ATP levels detected.

To experiment, cells were seeded at a density of 1.5 × 10^4^ cells per well in 96-well plates and treated with the tested compounds for 24 h. After treatment, the cells were washed with HBSS buffer and incubated with ATP-Glo™ detection cocktail. The luminescence was then measured using a Molecular Devices SpectraMax M2e Multi-Mode Microplate Reader with an emission wavelength of 560 nm. The percentage of ATP/viable cells was calculated relative to the control cells.

### Glutathione (GSH) measurement

Total intracellular GSH was measured following a modified mBCI method previously described [20]. mBCI is inherently nonfluorescent until conjugated, at which point it demonstrates a strong reactivity with various low molecular weight thiols, including glutathione. In this assay, cells were seeded according to the cell viability assay. After treatment, cells were washed with HBSS before adding 100 μL of mBCI test solution composed of IGEPAL (0.2%), glutathione S-transferase (0.5 unit/mL), and mBCI (30 μM) in HBSS, shaken for a minute, and incubated for 60 min at room temperature. Glutathione was then measured fluorometrically using a Molecular Devices SpectraMax M2e Multi-Mode Microplate Reader, with an excitation wavelength of 394 nm and an emission wavelength of 490 nm. The percentage of glutathione was calculated relative to the control cells.

### Lipid peroxidation measurement

As described in published articles [18, 19], we measured lipid peroxidation using the fluorescent probe C11-BODIPY 581/591. This fluorophore shows an increase in green fluorescence and a corresponding decrease in red fluorescence in response to lipid peroxidation. In this experiment, cells were seeded according to the cell viability assay and co-treated with the tested compounds and a ferroptosis inducer, which was prepared in media containing C11-BODIPY 581/591 at a final concentration of 2.5 μM. After treatment, the cells were washed once with PBS. We then supplemented the cells with PBS before measuring fluorescence. Red fluorescence was determined at 565/600 nm (excitation/emission), while green fluorescence was measured at 477/525 nm using a Synergy H1 microplate reader (BioTek Instruments, Winooski, USA). Lipid peroxidation was represented as the green to red fluorescence ratio of BODIPY 581/591 C11.

### Radical trapping antioxidant (RTA) activity assay

As described in published articles [18, 19], we utilized the ABTS radical scavenging assay to evaluate the RTA capabilities of the tested compounds. This colorimetric assay is commonly used to assess the antioxidant properties of various substances. In the first step of the assay, the ABTS reagent was mixed with potassium persulfate to generate free radicals. The resulting solution was then diluted with water to achieve an absorbance of 0.3 to 0.6 at 734 nm. In the second step, 100 μL of the ABTS reagent was combined with 100 μL of the tested sample in a 96-well microplate. This mixture was incubated at room temperature for 7 to 15 min before measuring the absorbance at 734 nm using a Molecular Devices SpectraMax M2e Multi-Mode Microplate Reader.

This assay measures the conversion of the dark blue ABTS radical cation into the colorless ABTS as a result of the action of antioxidants. DMSO at a 100% concentration served as the control. The ABTS radical scavenging properties of the tested compounds were then assessed using the following formula:

ABTS radical scavenging (%) = [(Control absorbance−Sample absorbance)/Control absorbance] × 100

### Statistical analysis

Statistical analysis was performed using Microsoft Excel 365 and GraphPad Prism 10 software for Windows (GraphPad Software, San Diego, CA, USA, www.graphpad.com). We employed non-linear regression analysis with a variable slope model to fit a logistic curve to the dose-response data, allowing us to determine the EC_50_ values along with their 95% confidence intervals. We did not report EC_50_ for dose-response data if the effectiveness is not beyond 50%. An unpaired *t* test was utilized to assess the significance between the two groups. One-way ANOVA was used for statistical comparisons, followed by Dunnett’s post hoc analyses when the ANOVA revealed significant differences among the groups. A *p*-value of less than 0.05 was considered statistically significant for all tests.

## Results

The hydroxyindole compounds tested were subjected to an initial pharmacokinetic evaluation using the SwissADME tool (Available: http://www.swissadme.ch/index.php). The results showed that, with the exception of serotonin and 5-HIAA, these hydroxyindole compounds can cross the blood-brain barrier (BBB) and exhibit similar physicochemical properties (Table 1).

**Table 1:** Predicted physicochemical properties of the tested hydroxyindoles obtained through in silico analysis.

Compounds	CAS number	MW (Desired CNS drug-likeness ≤ 360)	MLogP (Desired CNS drug-likeness ≤ 4.15)	BBB permeability
3-HI	480-93-3	133.15 g/mol	0.91	Yes
5-HI	1953-54-4	133.15 g/mol	0.91	Yes
6-HI	2380-86-1	133.15 g/mol	0.91	Yes
7-HI	2380-84-9	133.15 g/mol	0.91	Yes
Serotonin (5-HT)	153-98-0	176.22 g/mol	0.65	Yes
5-HIAA	54-16-0	191.18 g/mol	0.53	No

Next, we tested several hydroxyindole analogs, specifically 3-HI, 5-HI, 6-HI, and 7-HI, to evaluate their potential protective effects against ferroptosis in neuronal cultures. Erastin, an inhibitor of system Xc^–^ (cystine/glutamate antiporter), was used as a ferroptosis inducer. By inhibiting system Xc^–^, erastin leads to a depletion of cystine uptake, which suppresses glutathione synthesis and subsequently triggers ferroptosis [18, 19]. To confirm that the induced cytotoxicity was indeed driven by ferroptosis, we employed Lip-1, a standard ferroptosis inhibitor that functions through its RTA activity [19].

**Fig. 2:**
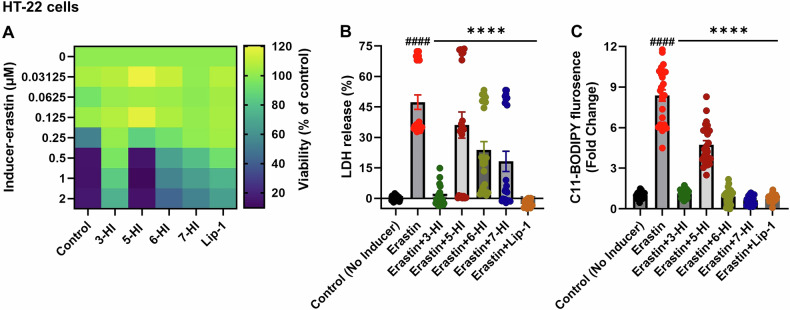
Hydroxyindoles protect against ferroptosis induced by erastin in HT-22 cells. Cell viability, cell death and C11-BODIPY assays were measured after co-treatment with various compounds for 22–24 h (**A**–**C**). The compounds included 20 µM of 3-HI, 6-HI, and 7-HI, as well as 1 µM of liproxsatatin-1 (Lip-1), all co-treated with the ferroptosis inducer erastin (**A**–**C**). Erastin concentration was 1 µM (B) and 0.5 µM (**C**). Cell viability was assessed using the MTT assay (A), while cell death was measured with an LDH release detection kit (**B**). Lipid peroxidation is expressed as the ratio of green to red BODIPY 581/591 C11 fluorescence (**C**). The data points represent the mean percentage of survival relative to untreated cells, expressed as mean ± SEM, with *n* = 9–24 from 2–3 independent experiments (**A**–**C**). An unpaired t-test was used to calculate the significance between the control and erastin (####*p* < 0.0001: control vs. erastin) (**B**, **C**). One-way ANOVA, followed by Dunnett’s post hoc analysis, was used to calculate statistical significance between erastin and treatment groups (****p < 0.0001: erastin vs. treatments) (**B**, **C**).

Our investigation demonstrated that hydroxyindole compounds (20 μM) significantly confer resistance to ferroptotic toxicity induced by erastin in HT-22 cells (Fig. 2). We showed that erastin causes cytotoxicity, as evidenced by a decrease in cell viability at higher concentrations. Among the tested hydroxyindole compounds, 3-HI exhibited the highest activity as a ferroptosis inhibitor, effectively protecting cells from the dose-dependent erastin toxicity. In contrast, the hydroxyindole compound 5-HI showed the lowest activity against ferroptosis (Fig. 2A). We further demonstrated that a single concentration of erastin causes cytotoxicity, as evidenced by elevated LDH release, and promotes lipid peroxidation, as indicated by a rise in C11-BODIPY 581/591 fluorescence. Both of these effects were significantly suppressed by the hydroxyindole compounds (Fig. 2B, C). Notably, 5-HI exhibited the lowest protective activity against erastin compared to other hydroxyindole compounds in both assays, including the LDH release and C11-BODIPY assays (Fig. 2B, C).

Serotonin and its metabolite 5-HIAA belong to the hydroxyindole class and are a specific example of 5-HI compounds. Given the role of serotonin in protecting against ferroptosis [11–14], we tested both serotonin and its metabolite 5-HIAA for their effects against ferroptosis in HT-22 cells. In addition to erastin, we used two other ferroptosis inducers, RSL3 and FINO2. RSL3 is one of the most potent inducers of ferroptosis; it inhibits GPX4 enzymatic activity, resulting in the failure of GPX4-mediated detoxification of lipid hydroperoxides, which leads to ferroptosis [18, 19]. FINO2, conversely, is a compound containing an endoperoxide with a 1,2-dioxolane structure [21]. Its mechanism of inducing ferroptosis is distinct from that of system Xc^–^ inhibitors or RSL3. In the case of FINO2, the presence of the endoperoxide moiety and a nearby hydroxyl group is crucial for its ferroptotic effects. FINO2 indirectly inhibits GPX4 enzymatic activity and directly oxidizes iron, resulting in extensive lipid peroxidation and ferroptosis [21].

**Fig. 3:**
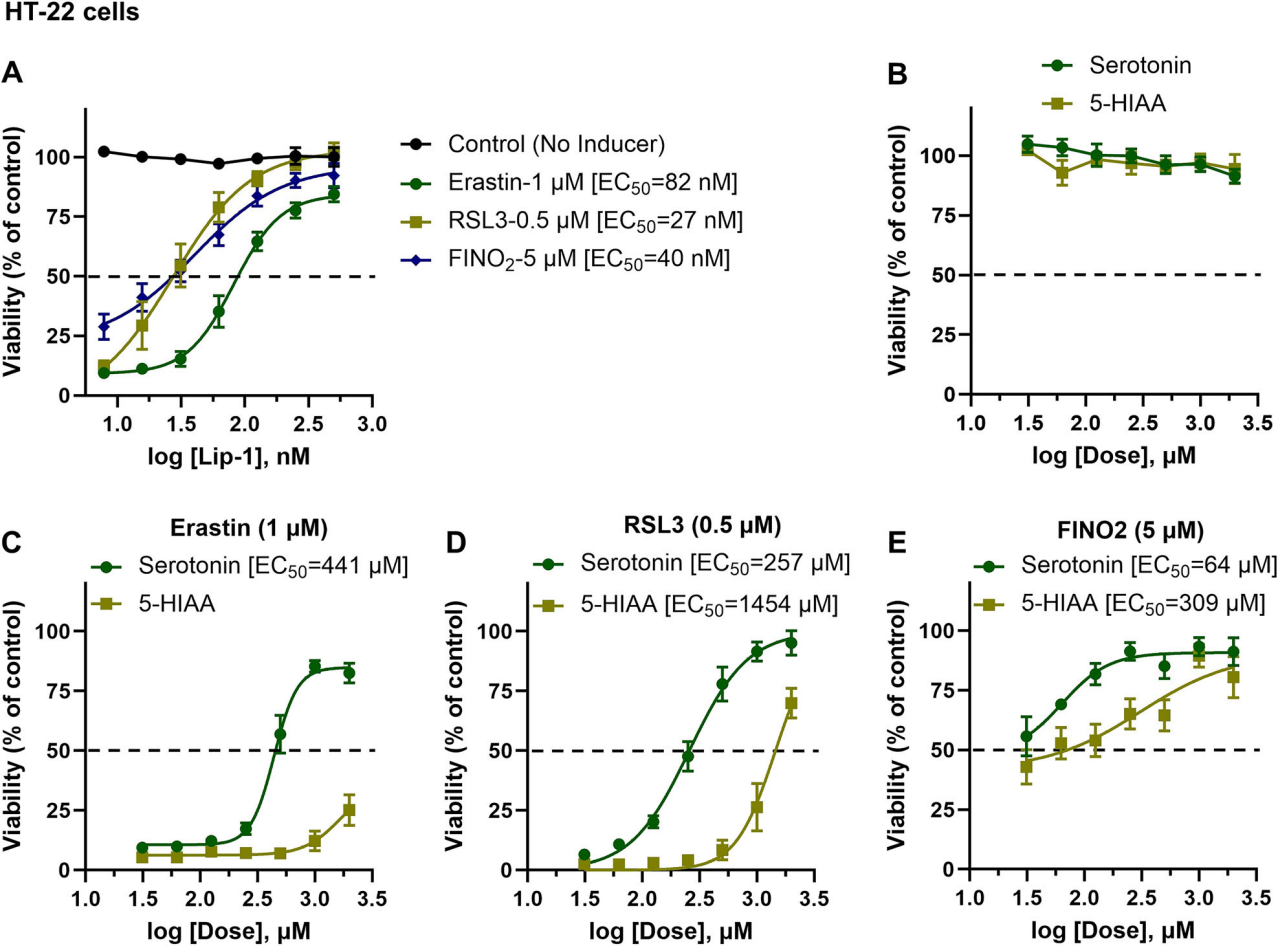
Anti-ferroptotic activity of serotonin and its metabolite 5-HIAA against various ferroptosis inducers in HT-22 cells. Cell viability was evaluated after 24 h of treatment with various compounds, both in the presence and absence of ferroptosis inducers (**A**–**E**). The viability was measured using the calcein AM assay. The data points represent the mean percentage of cell survival relative to untreated cells, expressed as ± SEM, with *n* = 9–12 from 3–4 independent experiments. We used non-linear regression analysis with a variable slope model to fit a logistic curve to the dose-response data in order to determine the EC_50_ and the 95% confidence interval (CI).

We first confirmed that a dose of inducers specifically causes ferroptosis by demonstrating that the standard ferroptosis inhibitor, Lip-1, can rescue HT-22 cells from death. The effective concentrations (EC_50_) for Lip-1 against erastin, RSL3, and FINO2 were determined to be 82 nM, 27 nM, and 40 nM, respectively (Fig. 3A). We subsequently tested serotonin and 5-HIAA and found no considerable toxicity of these compounds in HT-22 cells (Fig. 3B). Consistent with previous findings on serotonin’s protective role against ferroptosis [11–14], we discovered that serotonin and 5-HIAA mitigate the toxic effects of erastin, RSL3, and FINO2 in HT-22 cells (Fig. 3C–E). However, 5-HIAA exhibited mild protective activity, achieving less than 50% protection from erastin toxicity (Fig. 3C). Our results indicate that both serotonin (with EC_50_ values of 257 μM against RSL3 and 64 μM against FINO2) and 5-HIAA (with EC_50_ values of 1454 μM against RSL3 and 309 μM against FINO2) are relatively weak ferroptosis inhibitors compared to the standard inhibitor, Lip-1 (Fig. 3A and Fig. 3C–E), as well as hydroxyindole compounds such as 3-, 6, and 7-HI (Fig. 2). Due to the lower effectiveness of 5-HI and its analogs, including serotonin and 5-HIAA (Fig. 2 and Fig. 3C–E), we have decided to exclude them from further experimentation.

**Fig. 4:**
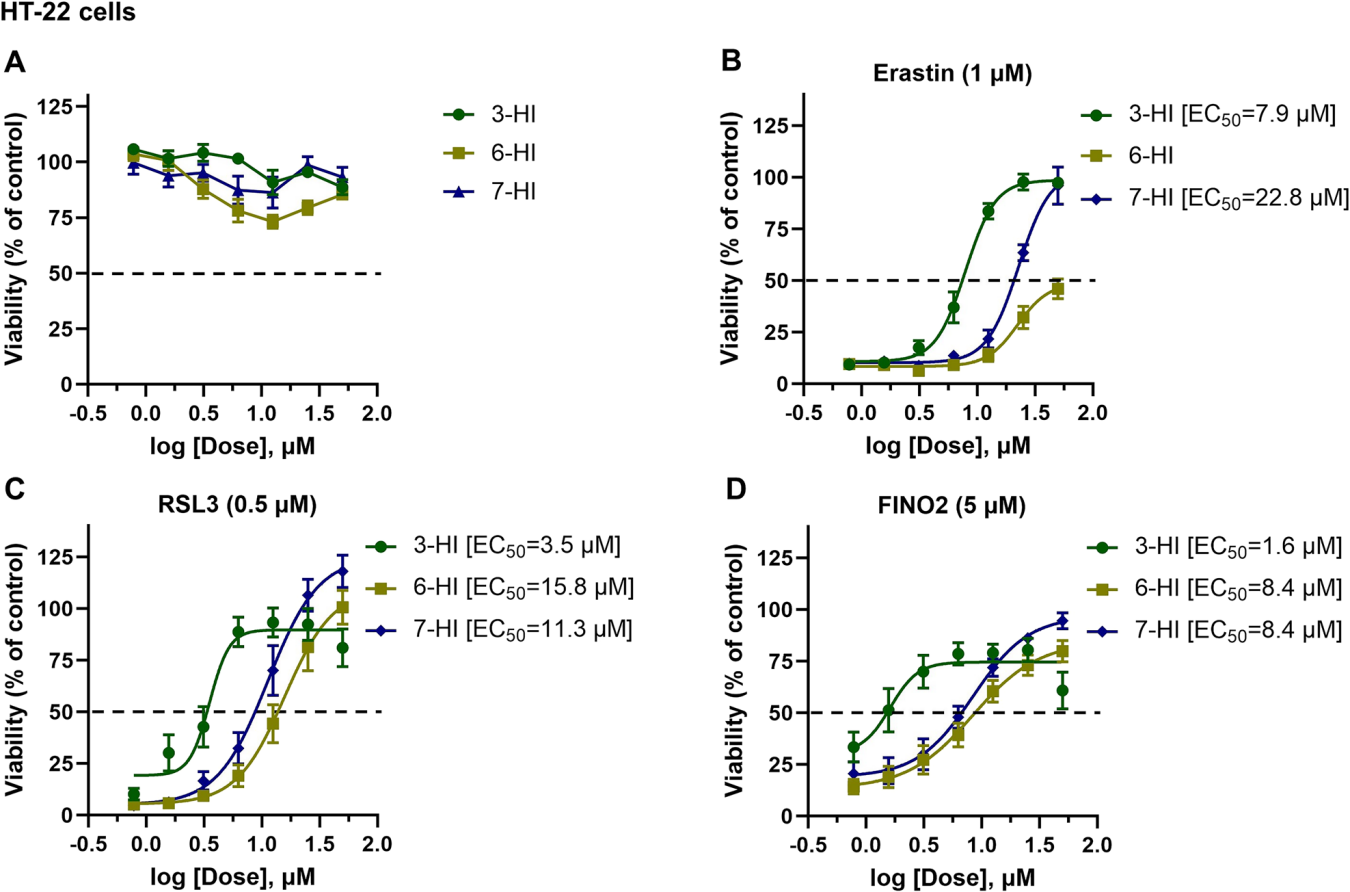
The comparative anti-ferroptotic activity of 3-, 6-, and 7-hydroxyindoles was evaluated against various ferroptosis inducers in HT-22 cells. Cell viability was evaluated after 24 h of treatment with various compounds, both in the presence and absence of ferroptosis inducers (**A**–**D**). The viability was measured using the calcein AM assay. The data points represent the mean percentage of cell survival relative to untreated cells, expressed as ± SEM, with *n* = 12–16 from 3–4 independent experiments. We used non-linear regression analysis with a variable slope model to fit a logistic curve to the dose-response data in order to determine the EC_50_ and the 95% confidence interval (CI).

Next, we assessed the potency of ferroptosis inhibition by hydroxyindoles against several inducers, specifically erastin, RSL3, and FINO2, in HT-22 cells. First, we confirmed the toxicity of hydroxyindoles and subsequently tested their potency for ferroptosis inhibition. 6-HI was found to be slightly toxic compared to 3-HI and 7-HI (Fig. 4A). Consistent with previous data (Fig. 2), we found that all tested hydroxyindoles, including 3-HI, 6-HI, and 7-HI, were protective against the toxicity caused by erastin, RSL3, and FINO2 (Fig. 4B–D); however, 6-HI showed less activity against erastin since maximum protection was around 50% (Fig. 4B). The potencies of ferroptosis inhibition were as follows: 3-HI (EC_50_ against erastin is 7.9 μM, RSL3 is 3.5 μM and FINO2 is 1.6 μM), 6-HI (EC_50_ against RSL3 is 15.8 μM and FINO2 is 8.4 μM), and 7-HI (EC_50_ against erastin is 22.8 μM, RSL3 is 11.3 μM and FINO2 is 8.4 μM). This indicates that 3-HI was the most potent ferroptosis inhibitor among the tested hydroxyindole compounds, and 7-HI did seem to be more potent than 6-HI (Fig. 4B–D).

**Fig. 5:**
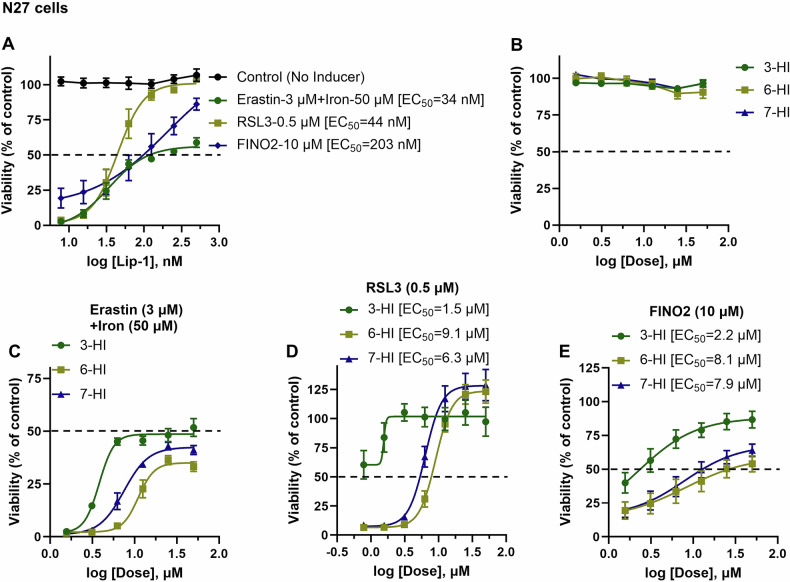
The comparative anti-ferroptotic activity of 3-, 6-, and 7-hydroxyindoles was evaluated against various ferroptosis inducers in N27 cells. Cell viability was evaluated after 24 h of treatment with various compounds, both in the presence and absence of ferroptosis inducers (**A**–**E**). Cell viability was assessed using the calcein AM assay. The data points represent the mean percentage of cell survival relative to untreated cells, expressed as ± SEM, with *n* = 9–12 from 3 independent experiments (**A**–**E**). We used non-linear regression analysis with a variable slope model to determine the EC_50_ with a 95% confidence interval (CI) to fit a logistic curve to the dose-response data.

We further evaluated the effectiveness of ferroptosis inhibition in N27 cells. Our experimental model used a combination of erastin with iron, RSL3, and FINO2 to induce ferroptosis. First, we confirmed that the standard ferroptosis inhibitor Lip-1 rescues cells from cytotoxicity caused by ferroptosis inducers, with an EC_50_ range against erastin+iron 34 nM, RSL3 44 nM and FINO2 203 nM (Fig. 5A). Notably, in our experiments, Lip-1 provided around 60% resistance to ferroptosis induced by the combination of erastin and iron (Fig. 5A).

Next, we assessed the hydroxyindole compounds in N27 cells and found no significant cytotoxicity associated with these compounds (Fig. 5B). Consistent with previous findings in HT-22 cells, we observed that 3-HI was the most effective inhibitor of ferroptosis induced by combined erastin and iron, RSL3 and FINO2, with an EC_50_ of 1.5 μM against RSL3 and 2.2 μM against FINO2 in N27 cells. In comparison, 6-HI had an EC_50_ of 9.1 μM against RSL3 and 8.1 μM against FINO2, and 7-HI had an EC_50_ of 6.3 μM against RSL3 and 7.9 μM against FINO2 (Fig. 5C–E), suggesting that 7-HI is more potent than 6-HI.

Our investigation aimed to uncover the protective mechanisms through which hydroxyindole compounds mitigate toxicity induced by various ferroptosis inducers. We examined the structure-activity relationship (SAR) of hydroxyindoles, building on existing literature highlighting the anti-ferroptotic properties of indole compounds attributed to their RTA characteristics [18]. RTA effects refer to the ability of antioxidants to interact directly with free radicals and neutralize them by forming stable compounds. This process helps to prevent further oxidative damage by interrupting the chain reactions caused by free radicals, effectively acting as a “chain-breaking” mechanism to stop the spread of oxidative harm.

**Fig. 6:**
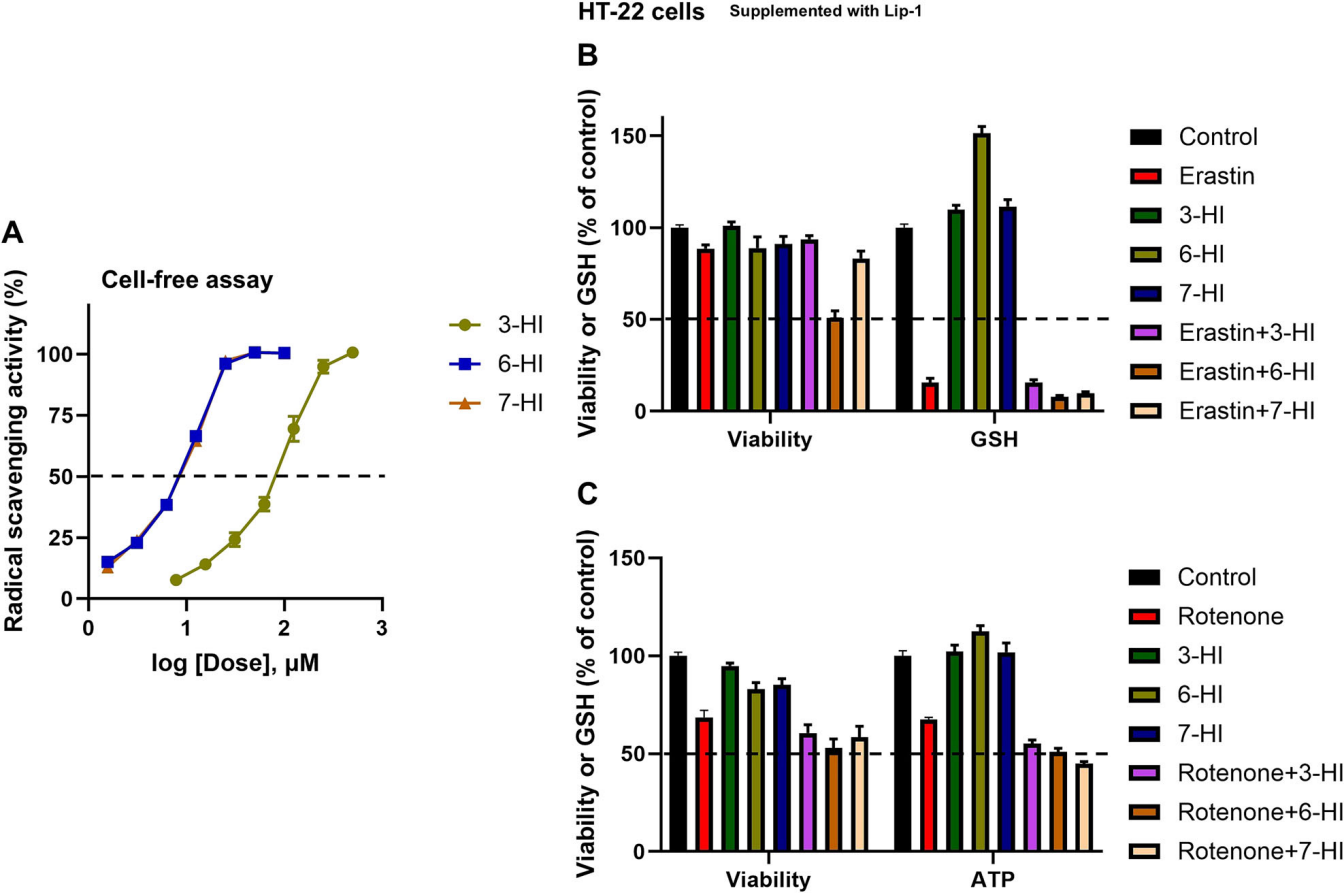
Radical trapping antioxidant (RTA) activity of hydroxyindoles and their impact on GSH regulation and ferroptosis-independent toxicity caused by rotenone. A cell-free ABTS assay was performed to evaluate the RTA properties, measured as the percentage of radical scavenging activity (**A**). Cell viability, ATP levels, and GSH concentration were assessed after co-treatment with various compounds for 24 h, supplemented with Lip-1 (1 µM) to prevent ferroptosis in HT-22 cells (**B**, **C**). The compounds used included 25 µM of 3-hydroxyindole (3-HI), 6-hydroxyindole (6-HI), and 7-hydroxyindole (7-HI). These treatments were co-treated with the inducers erastin (1 µM) and rotenone (20 µM) (**B**, **C**). The cell viability results indicated no ferroptotic cytotoxicity (**B**, **C**). Cell viability was evaluated using the calcein AM assay, ATP levels were measured with the ATP-Glo™ bioluminometric assay, and GSH levels were assessed using an mBCI-based assay (**B**, **C**). Data points represent the mean percentage survival compared to untreated cells ± SEM, with *n* = 12 from 3 independent experiments.

In our study, we used a cell-free ABTS assay to evaluate the antioxidant effects of various hydroxyindoles. Our results showed that all tested compounds exhibited increased radical scavenging (RTA) activity at higher doses (Fig. 6A). Notably, 3-HI displayed lower radical scavenging (RTA) activity in the cell-free ABTS assay, indicating less effective scavenging of ABTS radicals. However, it still demonstrated greater inhibition of ferroptosis in cell-based assays.

Furthermore, we investigated the impact of hydroxyindoles on glutathione levels and depletion in HT-22 cells, given that glutathione is essential for preventing ferroptosis. As previously mentioned, erastin inhibits glutathione synthesis, leading to ferroptosis. To determine whether hydroxyindole compounds enhance glutathione levels and/or rescue glutathione depletion, we treated cells with hydroxyindoles alone or erastin and hydroxyindole compounds while co-supplementing with the ferroptosis inhibitor Lip-1. This approach enabled us to examine the enhancement of glutathione or preservation of the erastin-induced loss of glutathione by the treatment in a condition where no ferroptotic cell death occurred. We first confirmed that cell survival was maintained despite glutathione depletion when cells were treated with erastin in the presence of Lip-1 (Fig. 6B). Interestingly, 6-HI increased glutathione levels independently but showed some toxicity when co-treated with erastin (Fig. 6B). While 6-HI was the only compound tested that elevated glutathione levels in HT-22 cells, it, along with other hydroxyindoles, did not preserve the reduction of glutathione caused by erastin (Fig. 6B). These findings suggest that the anti-ferroptotic effects of hydroxyindole compounds are primarily attributable to their RTA ability to combat ferroptosis.

We also tested hydroxyindoles to evaluate their effects against rotenone toxicity in HT-22 cells. Rotenone is a pesticide and neurotoxicant that inhibits mitochondrial complex I and is commonly used to induce dopaminergic toxicity in Parkinson’s disease research [22, 23]. Importantly, the toxicity caused by rotenone is independent of ferroptosis, as it can exert cytotoxicity even under conditions where ferroptosis is prevented (Fig. 6C). Our analysis also revealed that the loss of ATP following rotenone treatment is linked to its non-ferroptotic cytotoxic effects (Fig. 6C). Furthermore, our experiments demonstrated that the hydroxyindoles were unable to protect cells from rotenone toxicity, suggesting that their protective effects are specific to ferroptosis (Fig. 6C).

## Discussion

The emerging literature suggests ferroptosis and its role in the pathogenesis of neurodegenerative diseases [15, 16], highlighting the importance of identifying ferroptosis inhibitors for developing effective disease-modifying therapies. Hydroxyindole compounds are widespread and exhibit a variety of biological activities, making them significant in medicinal applications. Therefore, we investigated their potential effects on modulating ferroptosis.

While prior studies have shown that certain hydroxyindole analogs, particularly those containing the 5-HI scaffold, can counteract cellular stress, including ferroptosis [11–14], the comparative effects of various hydroxyindoles on ferroptosis have not been fully explored. Our findings revealed that several hydroxyindole compounds—such as 3-HI, 5-HI, 6-HI, 7-HI, serotonin, and 5-HIAA—can desensitize neuronal cells to ferroptosis. When we assessed their efficacy in inhibiting ferroptosis in HT-22 cells, we established the following order of potency: 3-HI > 7-HI > 6-HI > 5-HI > serotonin > 5-HIAA. However, these compounds were found to be less potent than the standard ferroptosis inhibitor Lip-1. We summarized that these compounds inhibit ferroptosis, likely due to their intrinsic RTA activity. This finding aligns with prior studies on indole compounds that also inhibit ferroptosis through their RTA functions [11, 18, 24]. Our study enhances the understanding of hydroxyindole biology in relation to ferroptosis.

A hydroxyl group at the 3 position enhances the anti-ferroptotic effect of hydroxyindole. Our findings indicate that 3-HI is the most potent inhibitor of ferroptosis. This observation is consistent with previous research that identified 3-HI as a potent inhibitor of amyloid fibril formation and its associated cytotoxicity [4]. This compound could be further tested in models to explore its potential neuroprotective effects against neuronal injury related to amyloid beta and ferroptosis, particularly in neurodegenerative diseases such as Alzheimer’s disease.

Research indicates that, in addition to 3-HI, 4-HI effectively inhibits amyloid fibril formation and its associated cytotoxicity [4]. Psilocin (4-hydroxy-N,N-dimethyltryptamine), an analog of 4-HI, is a substituted tryptamine alkaloid and a serotonergic psychedelic found in many mushroom species. Studies have shown that psilocin binds directly to the TrkB receptor with affinities 1,000 times greater than those of other antidepressants, promoting neuroplasticity in mice [25]. Another study also demonstrated that psilocin increases brain-derived neurotrophic factor (BDNF) levels mediated by the serotonin 2 A receptor, further enhancing plasticity in human cortical neurons [26]. Since we did not evaluate these compounds in our study, psilocin and 4-HI could be tested for their potential anti-ferroptotic and neuroprotective effects.

We conducted an in-depth evaluation of a specific hydroxyindole, 5-HI, which is a neuroactive metabolite derived from L-tryptophan. The 5-HI scaffold is present in several biological molecules in humans, including melanin and serotonin. In our experiments, we also assessed the serotonin metabolite 5-HIAA. Our findings identified 5-HI and 5-HIAA as suppressors of ferroptosis, aligning with previous research that found serotonin protects against ferroptosis [11], and 5-HI protects against oxidative damage and cytotoxicity induced by glutamate and tert-butylhydroperoxide [3, 5, 6]. Since we found that 5-HI and its analogs, serotonin and 5-HIAA, showed lower activity than other hydroxyindoles, the hydroxyl group at the 5 position on the indole ring may reduce the anti-ferroptotic effect of hydroxyindole compounds. Another significant finding was that serotonin and 5-HIAA displayed lower activity than their parent compound, 5-HI. This indicates that additional groups, such as 2-aminoethyl or acetic acid at the 3 position on serotonin or 5-HIAA, influence their activity regarding ferroptosis. This highlights the complex interplay of structural modifications in the modulation of ferroptosis.

5,7-dihydroxytryptamine hydrobromide, another 5-HI and serotonin analog, can deplete serotonin and is toxic to serotonergic neurons [27, 28]. Given that this compound has two hydroxyl groups, whether it protects against or sensitizes to ferroptosis is unclear. If this compound sensitizes to ferroptosis, it is crucial to determine whether this effect results from serotonin depletion.

Finally, we have discovered an additional function of 6-HI beyond its role as an RTA; specifically, it enhances glutathione levels. However, this function is less likely to be responsible for its ability to combat ferroptosis, as other hydroxyindole compounds, such as 3-HI and 7-HI, provide superior resistance against ferroptosis without possessing this enhancement function. In addition, the reduction of glutathione caused by erastin showed a similar pattern when cells were treated with either 6-HI or other hydroxyindole compounds in a condition where ferroptosis was unlikely to occur.

Since the mechanism by which 6-HI increases glutathione levels remains unclear, further investigation is needed to determine whether it directly influences the transporters or enzymes that regulate glutathione synthesis. Given that 6-HI has functions beyond RTA, this compound may serve as a scaffold for synthesizing additional lead compounds that could exhibit improved pharmacokinetic and pharmacodynamic properties.

## Conclusion

**Fig. 7:**
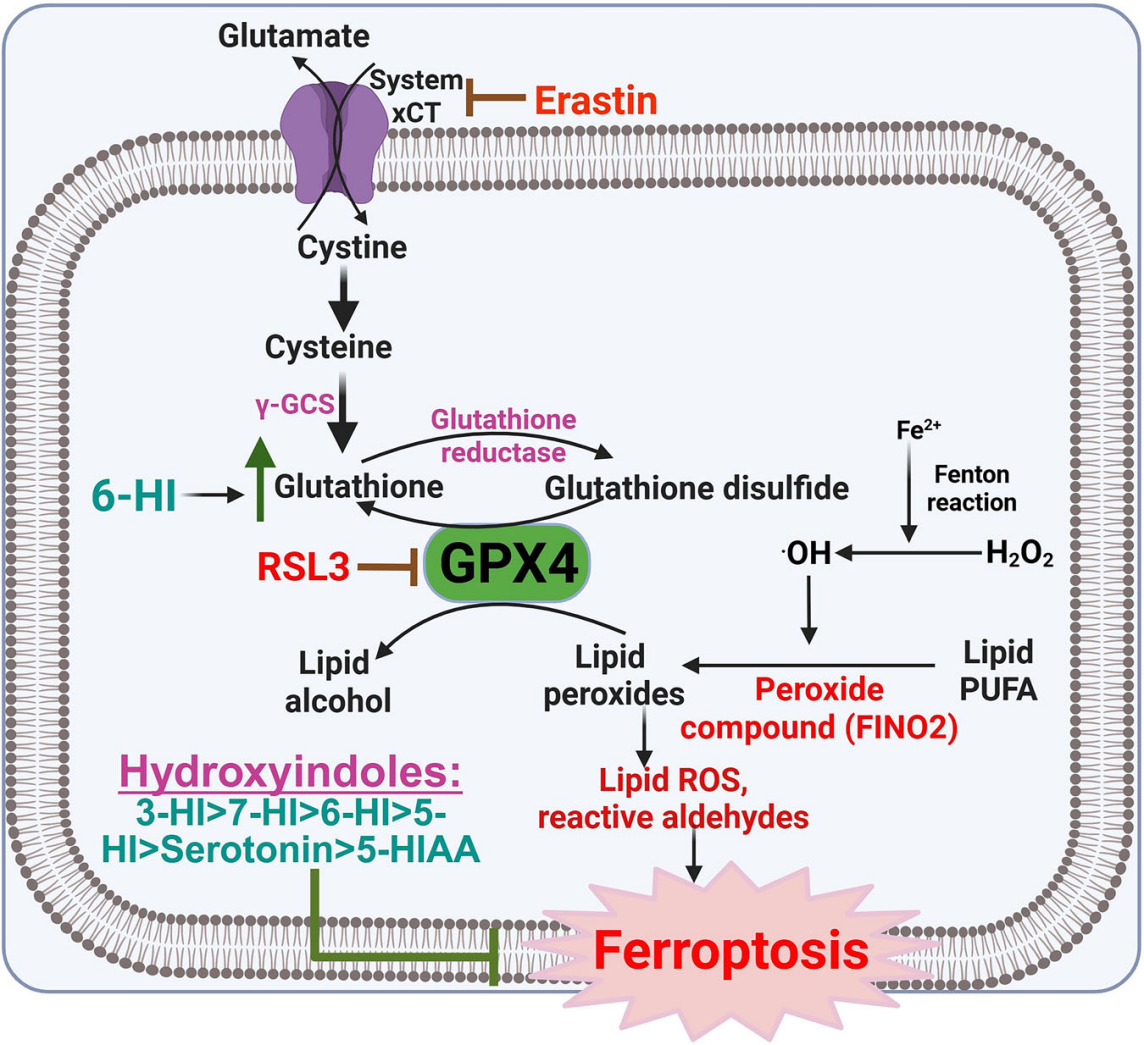
Summary of the anti-ferroptotic effects of hydroxyindoles. Erastin, RSL3, and FINO2 promote ferroptosis by increasing the production of lipid peroxides. Among the hydroxyindoles, 3-hydroxyindole (3-HI) is the most effective inhibitor of ferroptosis, outperforming other hydroxyindoles such as 6-hydroxyindole (6-HI), 7-hydroxyindole (7-HI), serotonin, and the serotonin metabolite 5-hydroxyindoleacetic acid (5-HIAA). Interestingly, 6-HI can increase glutathione levels, but this enhancement likely does not contribute to its anti-ferroptotic function.

This study demonstrated that hydroxyindoles can protect neuronal cultures from ferroptosis and identified a potential mechanism behind this protective effect. Among the hydroxyindoles tested, 3-HI was found to be the most effective inhibitor of ferroptosis in neuronal cells, providing strong protection against erastin, RSL3, and FINO2. The potency of these compounds in HT-22 cells is ranked as follows: 3-HI > 7-HI > 6-HI > 5-HI > serotonin > 5-HIAA (Fig. 7). Notably, we observed that 6-HI specifically increases glutathione levels (Fig. 7). Our experimental results suggest that 3-HI, 6-HI, and 7-HI warrant further investigation for their potential role in neuroprotective therapies to combat ferroptosis.

## Data Availability

All raw data will be available upon a reasonable request.
